# Supplementary material for: Heat Shock Protein and Disaggregase Influencing the Casein Structuralisation
Source: Int J Mol Sci. 2025 Jul 1;26(13):6360. doi: 10.3390/ijms26136360 (PMC12249863; doi:10.3390/ijms26136360)
Supplement: Supplementary file 1 [file ijms-26-06360-s001.zip › ijms-3677407-supplementary.pdf]

SUPPLEMENTARY MATERIALS

HEAT-SHOCK PROTEIN AND DISAGGREGASE INFLUENCING THE CASEIN STRUCTURALISATION

Roterman I[1], Stapor K[2]. Dułak D[3], Konieczny L[4].

RD and K scale as a measure of assessing the degree of incorrectly folded proteins

The source of the set of incorrectly folded structures may be the set of models provided by the CASP project participants. The evaluation of the submitted models may provide an overview of the possibilities of misfolded proteins. The T1266-D1 target from the CASP16 edition, was randomly selected. The project provided 62 models evaluated according to the GDT_TS classification (Table S1) [1].

Table S1. The results of model evaluations for the T1266-D1 target in the CASP16 project. The following are given: the position in the ranking list according to the CASP16 project evaluation, the model identifier, the RD value, the absolute value of the RD difference of the target and the model, the GDT_TS evaluation (the evaluation scale according to the CASP project) and the value of the K parameter for each model. The first position (0) in bold contains the parameter values for the target.

Nr	TARGET/MODEL	RD	D RD	GDT_TS	K
0	**T1266_1-D1**	**0.643**	**0.0**		**0.8**
1	T1266TS163_1-D1	0.603356	0.039645	92	0.7
2	T1266TS358_1-D1	0.616593	0.026407	91.3	0.7
3	T1266TS286_1-D1	0.599682	0.043319	91	0.7
4	T1266TS269_1-D1	0.608158	0.034843	90.6	0.7
5	T1266TS059_1-D1	0.605437	0.037563	90.1	0.7
6	T1266TS052_1-D1	0.613347	0.029653	90	0.7
7	T1266TS164_1-D1	0.603749	0.039251	89.8	0.7
8	T1266TS079_1-D1	0.60283	0.04017	89.5	0.7
9	T1266TS293_1-D1	0.60283	0.04017	89.5	0.7
10	T1266TS221_1-D1	0.601535	0.041465	89.2	0.7
11	T1266TS388_1-D1	0.611341	0.031659	89.2	0.7
12	T1266TS022_1-D1	0.597644	0.045356	89	0.7

13	T1266TS456_1-D1	0.598213	0.044787	88.8	0.7
14	T1266TS369_1-D1	0.602382	0.040618	88.2	0.7
15	T1266TS110_1-D1	0.603852	0.039148	87.9	0.7
16	T1266TS198_1-D1	0.610102	0.032899	87.9	0.7
17	T1266TS462_1-D1	0.603852	0.039148	87.9	0.7
18	T1266TS345_1-D1	0.611707	0.031293	87.7	0.7
19	T1266TS465_1-D1	0.607919	0.035081	87.7	0.7
20	T1266TS419_1-D1	0.599388	0.043612	87.5	0.7
21	T1266TS112_1-D1	0.607914	0.035086	87.3	0.7
22	T1266TS017_1-D1	0.603185	0.039815	87.1	0.7
23	T1266TS031_1-D1	0.603816	0.039184	87.1	0.7
24	T1266TS196_1-D1	0.601545	0.041455	87.1	0.7
25	T1266TS261_1-D1	0.583604	0.059396	87	0.6
26	T1266TS272_1-D1	0.599294	0.043706	86.7	0.7
27	T1266TS023_1-D1	0.599585	0.043416	86.5	0.7
28	T1266TS145_1-D1	0.599585	0.043416	86.5	0.7
29	T1266TS331_1-D1	0.608254	0.034746	86.4	0.7
30	T1266TS019_1-D1	0.602711	0.040289	86	0.7
31	T1266TS148_1-D1	0.614178	0.028822	85.9	0.7
32	T1266TS264_1-D1	0.614178	0.028822	85.9	0.7
33	T1266TS312_1-D1	0.614178	0.028822	85.9	0.7
34	T1266TS015_1-D1	0.600297	0.042703	85.6	0.7
35	T1266TS375_1-D1	0.623487	0.019514	84.8	0.8
36	T1266TS014_1-D1	0.60351	0.03949	84.6	0.7
37	T1266TS208_1-D1	0.62829	0.01471	84.4	0.8
38	T1266TS311_1-D1	0.597534	0.045466	84.2	0.7
39	T1266TS304_1-D1	0.619705	0.023295	84.2	0.7
40	T1266TS425_1-D1	0.602992	0.040008	83.9	0.7
41	T1266TS139_1-D1	0.595292	0.047708	83.5	0.7
42	T1266TS241_1-D1	0.621533	0.021467	83.3	0.7
43	T1266TS147_1-D1	0.595289	0.047712	82.6	0.7
44	T1266TS319_1-D1	0.618225	0.024775	81.3	0.7
45	T1266TS051_1-D1	0.613728	0.029272	79.7	0.7

46	T1266TS314_1-D1	0.62382	0.01918	79.5	0.8
47	T1266TS294_1-D1	0.626262	0.016738	78.2	0.7
48	T1266TS361_1-D1	0.571648	0.071352	74.3	0.6
49	T1266TS120_1-D1	0.572918	0.070082	73.9	0.6
50	T1266TS267_1-D1	0.687765	0.044765	58.8	1.1
51	T1266TS235_1-D1	0.645477	0.002477	56.6	0.8
52	T1266TS122_1-D1	0.728873	0.085873	55.2	1.5
53	T1266TS287_1-D1	0.740298	0.097298	55.2	1.5
54	T1266TS075_1-D1	0.684915	0.041915	54.4	1.1
55	T1266TS284_1-D1	0.684915	0.041915	54.4	1.1
56	T1266TS301_1-D1	0.684915	0.041915	54.4	1.1
57	T1266TS475_1-D1	0.684915	0.041915	54.4	1.1
58	T1266TS040_1-D1	0.71154	0.06854	53.7	1.1
59	T1266TS167_1-D1	0.595587	0.047413	51.2	0.7
60	T1266TS450_1-D1	0.710833	0.067833	43.3	1.3
61	T1266TS212_1-D1	0.710524	0.067524	41.9	1.1
62	T1266TS105_1-D1	0.76849	0.12549	6.78	1.4

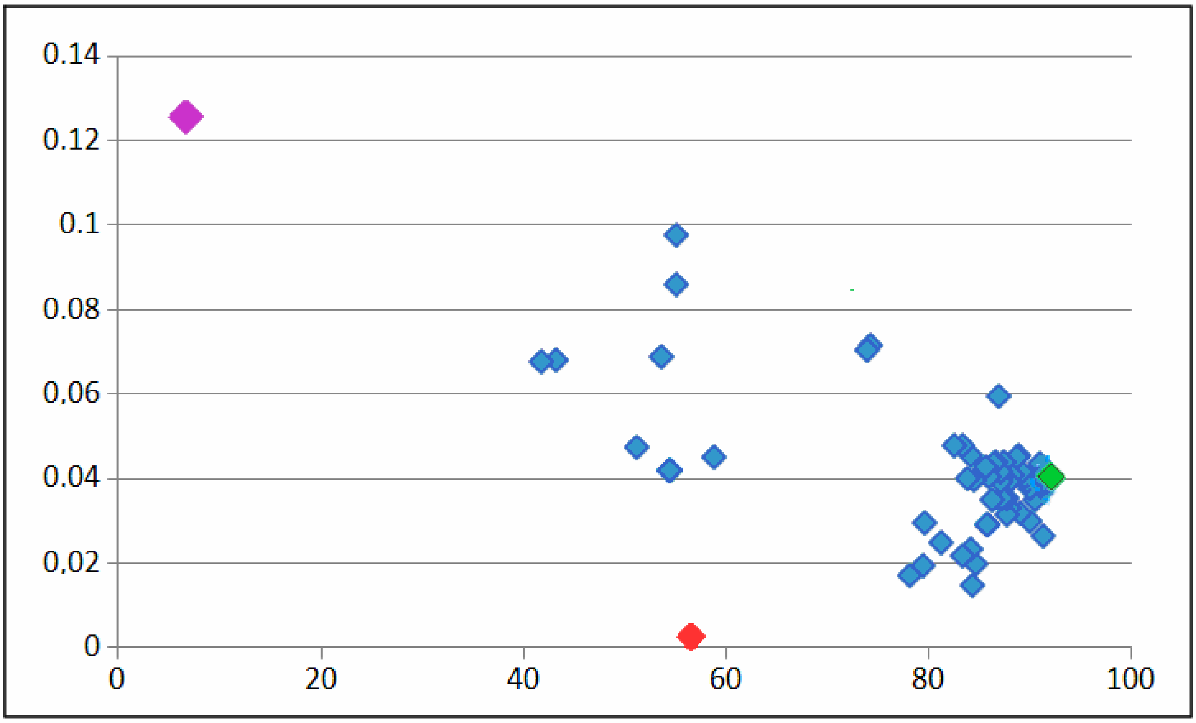

Figure S1. Assessment of the agreement between a model and the target: the absolute value of the difference (RD(target)-RD(model)) (vertical axis) in relation to the GDT_TS scale

(horisontal axis). The best model (green - #163). red (model #235) and purple (model #107 – the lowest one) discussed in details.

The results in Fig. S1 reveal a linear dependence between GDT_TS scale score used in the CASP project (the higher the value of this parameter, the higher the model's agreement with the target) and the differences in the status defining the hydrophobicity distribution in the designed model (the absolute value of the difference RD(target)-RD(model)).

The correlation coefficient determined for the set of results (Fig. S1) is -0.617.

The correlation coefficient between the GDT_TS score and the difference in K vs. K for the target is -0.75. The relationships between the scores according to the CASP project standards and the difference expressed using parameters based on the FOD-M model show high agreement. This means that reproducing the hydrophobicity distribution (RD parameter) and the influence of external factors on the protein (model) structuring (K parameter) determines correct folding.

The detailed analysis of the models highlighted in Fig. S1 discussed below aims to demonstrate the validity of the structure assessment based on the parameters based from FOD-M model.

The highlighted model #163 (green point in Fig. S1) assessed the best one on the scale used in the CASP project (GDT_TS) shows very high agreement compared to the assessment based on the hydrophobicity distribution. The comparison of the T and O distributions for the model against the analogous target distributions reveals no differences (Fig. S1.)

The highlighted model #50 (red on Fig. S1) is discussed to explain the reasons for the low assessment on the GDT_TS scale against the very positive assessment on the RD value scale (the highest agreement of the RD value distributions for this model).

Fig. S2. visualizes the relations of the T, O and M distributions (for the appropriate value of the K parameter) for the highlighted models (Fig. S1).

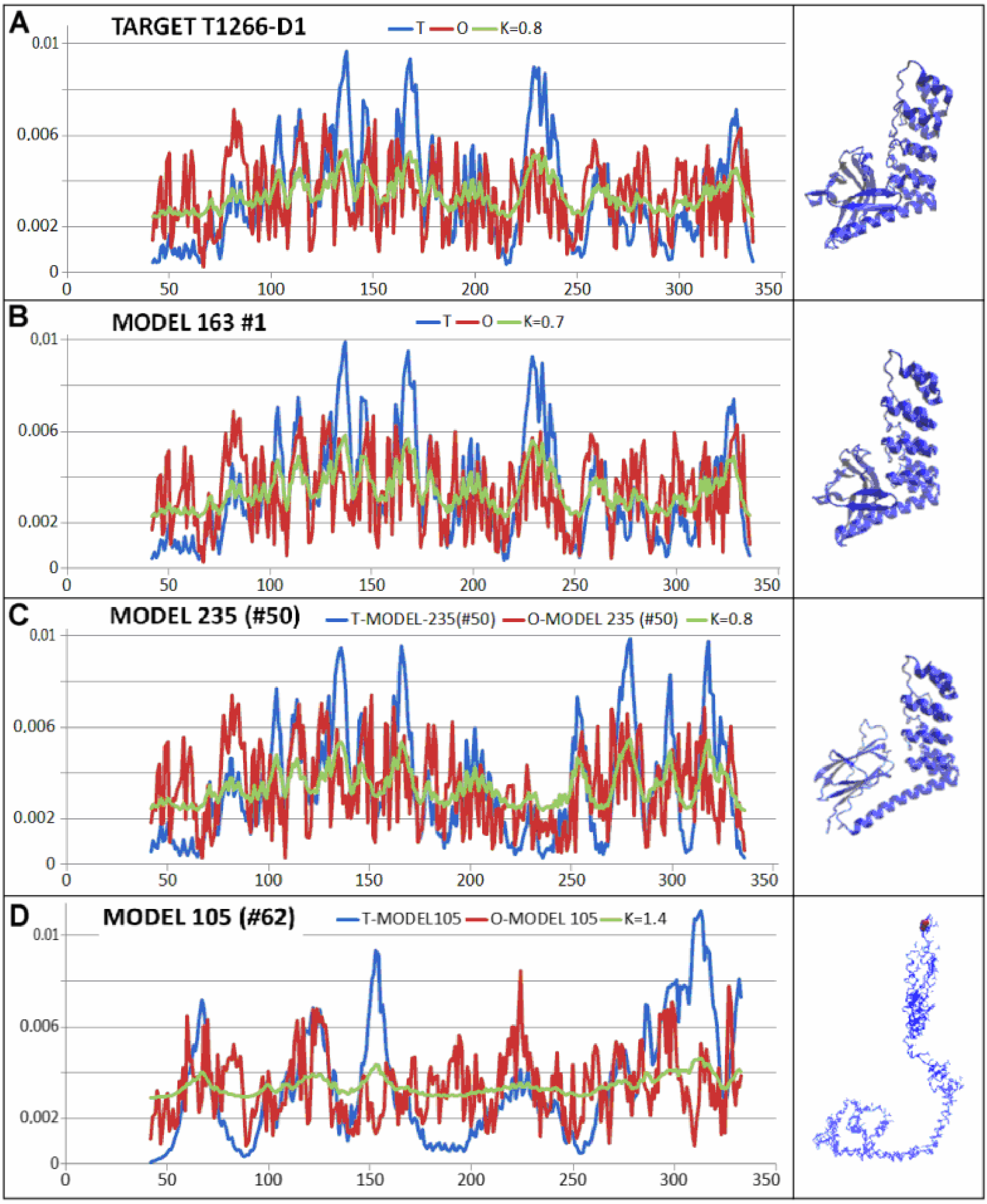

Figure S2. Profiles T, O and K for the corresponding K value given in the legend) along with the 3D presentation of the structures, respectively. A – target T1266, B – model#163 – the highest rated structure according to the GDT_TS scale, C – model #235 –the lowest difference in the model's RD value from the target's RD, D – model #105 – lowest GDT_TS value on the ranking list.

The highest rated model (model#163) shows a low difference in the RD parameter value (0.039) with K equal to 0.8 for the target and 0.7 for the model. The assessment of these

differences from the point of view of the FOD-M model is negligible. It can therefore be stated that the model and the target represent very similar structures. If we assume that the distribution of residues showing a different level of hydrophobicity Oi compared to the expected level Ti determines the location of the area associated with biological activity, then the positions of such residues in the model coincide with analogous positions in the target. The high agreement of model #163 (using the FOD-M based criteria) with the target is also revealed by the set of T profiles (Fig. S3.A) for the target and the model, and the high agreement of the O distribution for the target and the model (Fig. S3.B).

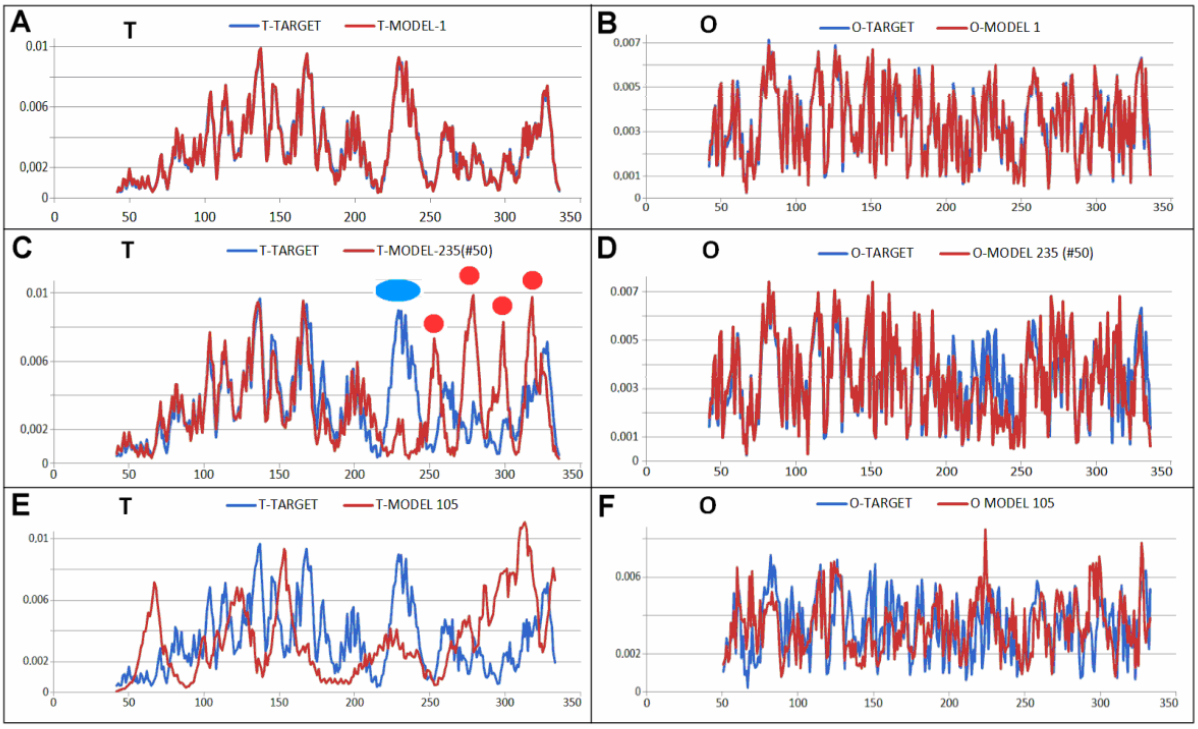

Figure S3. Comparable analysis of T and O distributions of models: A – The best model (#163) – comparison of T distributions of the target and the model. B - – The best model (#163) – comparison of O distributions of the target and the model. C - – The model (#235) – comparison of T distributions of the target and the model with the minimum difference in RD values. Highlighted positions – blue ellipse – segment building the concentration of hydrophobicity in the target, red circles – residues involved in the construction of hydrophobic concentration in model #235. Residues highlighted with blue oval and red circles – residues involved in the construction of hydrophobic concentrations presented as green in the 3D presentation – Figure S1. D – The model (#235) – comparison of the O distributions of the target and the model with the minimum difference in RD values. E - – The model (#105) – comparison of the T distributions of the target and the model with the largest discrepancy between the structure of the target and the model. F – The model (#105) – comparison of the O distributions of the target and the model with the largest discrepancy between the structure of the target and the model.

Model #235 is very interesting due to the almost perfect reproduction of distributions on the RD scale, while there is a significant difference for K. The model reproduces the concentration of hydrophobicity. Which was achieved in the model using different residues (Fig. S3.C). The residues highlighted in Fig. S3.C with a red dot build hydrophobicity concentrations in a similar spatial location, while in the target this center is built from different residues (Fig. S3.C – blue line). The final effect in the form of the appropriate RD value is almost identical. However, the value of the K parameter shows differences. The O distributions turn out to be highly convergent (Fig. 3S.D) with the exception of this section, which incorrectly generated the locations of hydrophobicity concentration (Fig. S4).

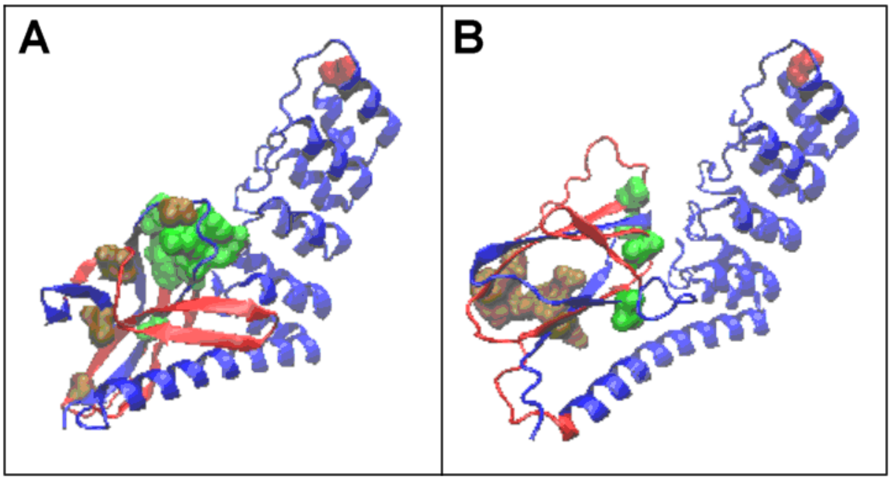

Figure S4. 3D structure of target and model. A – target T1266. B – model #235.The following were distinguished in the picture: red space filling – N-terminal position, navy blue part – part with identical T and O distribution in both compared structures. Red fragment – C-terminal part starting from position 210 with a different structure; green – residues engaged in hydrophobicity concentration. Ochre space filling – parts alternately not engaged in hydrophobicity concentration. Green and one structural form residues changed to ochre in the second one. Thre green residues on A are ochre on B and vice versa.

The 3D presentation visualizes the different location of residues highlighted as green space filling and the different involvement of residues in the construction of the hydrophobicity concentration center (residues green). This situation seems to be analogous to the example discussed in [19], where the exposure of residues on the surface of the final WT structure was ensured by the previous interaction of these residues with the inner surface of the chaperonin. This interaction determined the location of residues that formed the interface for the final construction of the complex ensuring the planned biological function of reovirus mu1/σ3w.

The $105 model shows significant differences in the values of the RD and K parameters. Fragments expected as hydrophobic core builders (104-114, 129-152, 162-179, 195-202 and 223-241) show a deficit of hydrophobicity. On the other hand, the section 283-320 representing a very high level of hydrophobicity is located on the surface, where low levels of hydrophobicity are expected. The very high value of K = 1.4 indicates theoretically the possibility of generating such a structure in a very changed environment - according to the interpretation based on the FOD-M model.
Model$105 characterised by T and O profiles clearly represent the significantly different construction of T distribution. Folding process following this distribution produces the misfolded form of the protein (Fig.S3.E and Fig.S3.F and Fig. S2.D).

The models discussed here are treated as examples of incorrectly formed spatial structures. The aim is to demonstrate the possibility of expressing structural correctness using FOD-M model parameters. It also poves the the necessity to recreate the hydrophobicity distribution as the important factor for structure and biological activity of protein under consideration.

References

1. Available online: https://predictioncenter.org/casp16/results.cgi?view=tables&target=T1266-D1& (accessed on 15 May 2025).
